# Supplementary material for: The Enzyme 15-Hydroxyprostaglandin Dehydrogenase Inhibits a Shift to the Mesenchymal Pattern of Trophoblasts and Decidual Stromal Cells Accompanied by Prostaglandin Transporter in Preeclampsia
Source: Int J Mol Sci. 2023 Mar 7;24(6):5111. doi: 10.3390/ijms24065111 (PMC10049104; doi:10.3390/ijms24065111)
Supplement: Supplementary file 1 [file ijms-24-05111-s001.zip › Supplementary Table S1.pdf]

Table S1. Clinical features of PE and control subjects

Characteristics	PE(n=8)	NA(n=8)	p
Age at gestation (years)	32.45±3.64	31.73±4.21	0.5867
Gestational age (weeks)	34.58±3.04	36.58±4.33	0.1180
Gravidity	3.12±0.71	2.73±0.56	0.0761
Parity	0.42±0.59	0.63±0.44	0.2344
BMI (kg/m^2)	28.66±0.84	29.51±2.37	0.1606
SBP (mmHg)	163.18±25.06	109.41±19.40	<0.0001
DBP (mmHg)	97.34±12.25	71.25±14.38	<0.0001
Cesarean section (%)	100	100	1
