# Supplementary figures and images for: The Enzyme 15-Hydroxyprostaglandin Dehydrogenase Inhibits a Shift to the Mesenchymal Pattern of Trophoblasts and Decidual Stromal Cells Accompanied by Prostaglandin Transporter in Preeclampsia
Source: Int J Mol Sci. 2023 Mar 7;24(6):5111. doi: 10.3390/ijms24065111 (PMC10049104; doi:10.3390/ijms24065111)

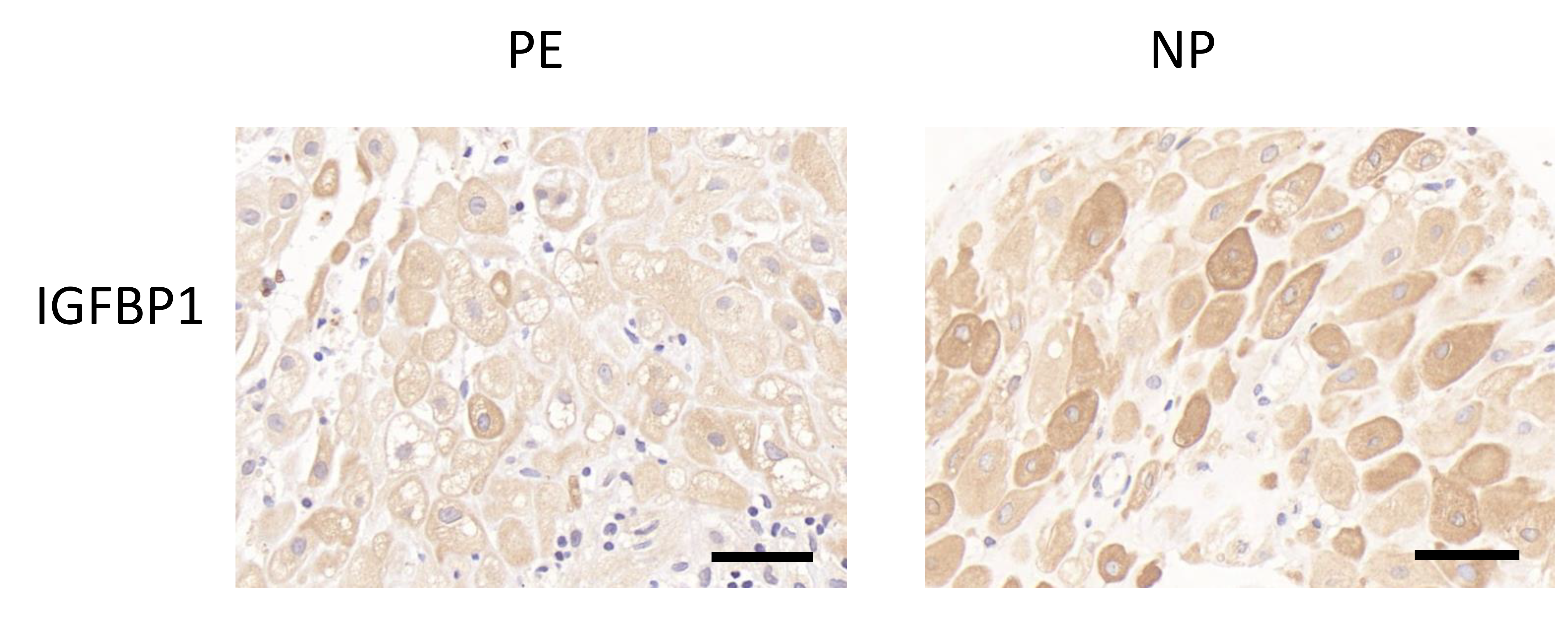

Supplement: Supplementary file 1 [file ijms-24-05111-s001.zip › Figure S1.tif]

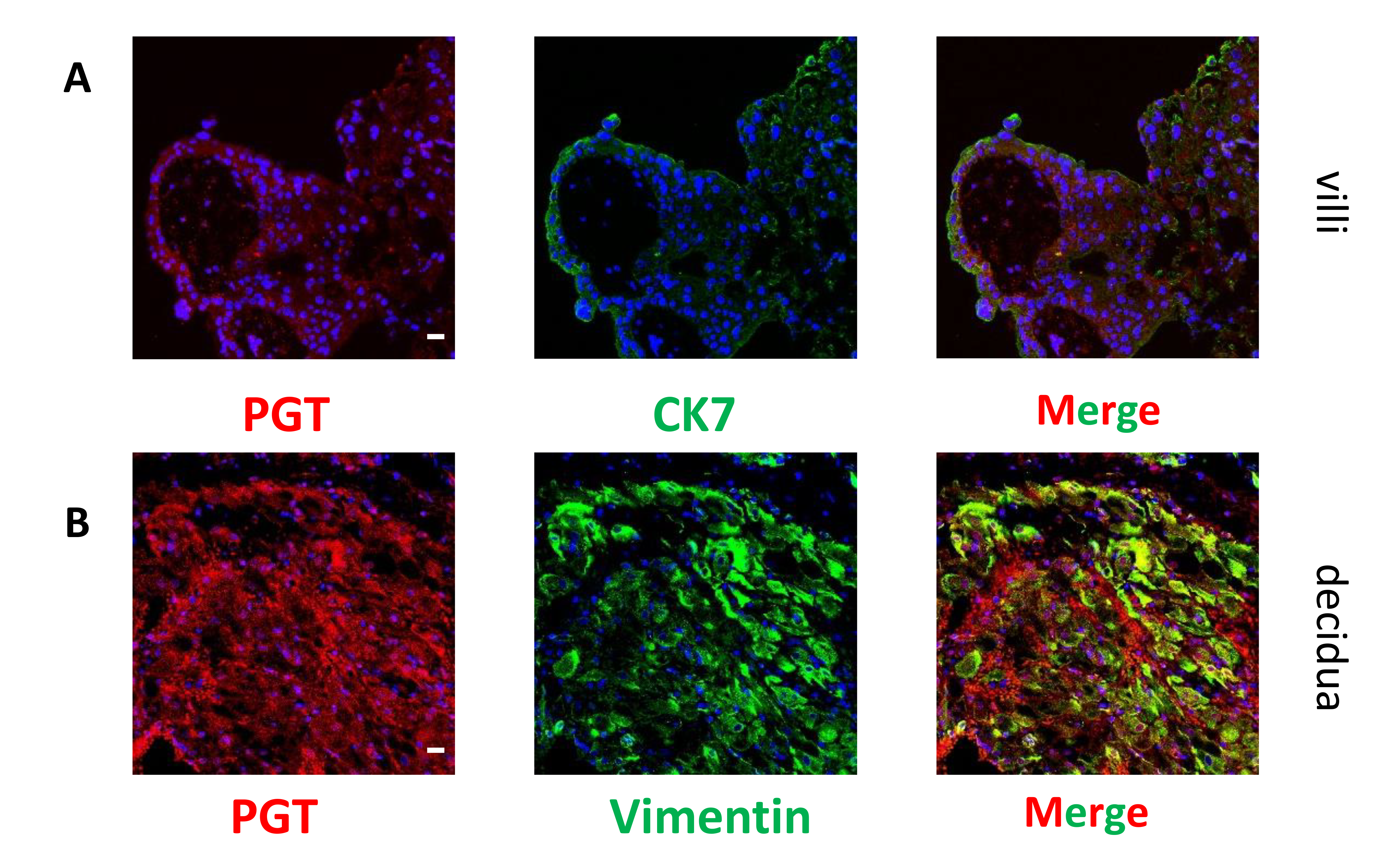

Supplement: Supplementary file 1 [file ijms-24-05111-s001.zip › Figure S2.tif]

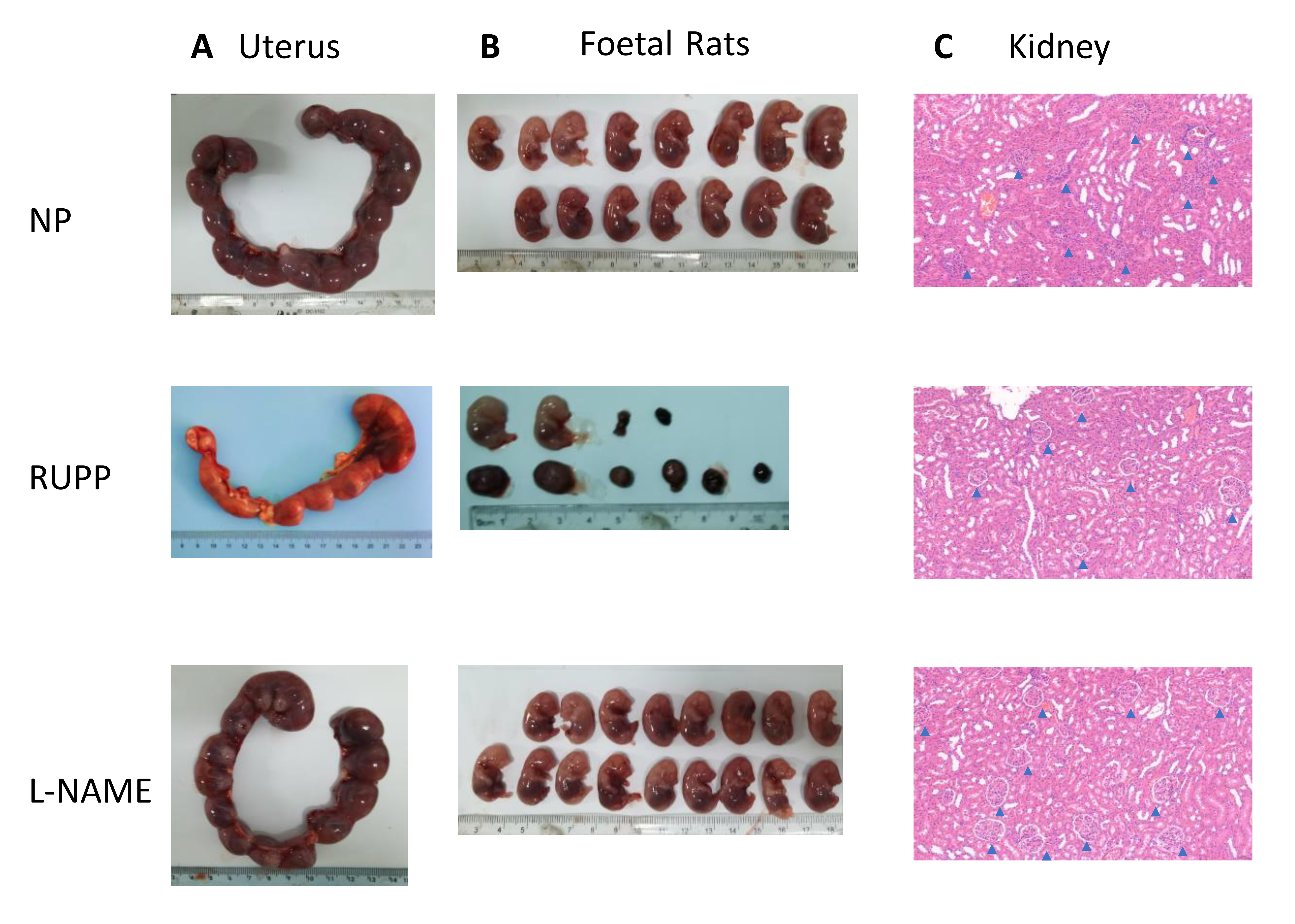

Supplement: Supplementary file 1 [file ijms-24-05111-s001.zip › Figure S3.tif]
